# Liveborn trisomy 22: a case report

**DOI:** 10.3389/fped.2026.1920011

**Published:** 2026-10-14

**Authors:** Sofia Jares Baglivo, Megan P. Morand, Gopalrao V. N. Velagaleti, Sunil K. Jain, Erin Cooney

**Affiliations:** 1 Department of Pediatrics, University of Texas Medical Branch, Galveston, TX, United States; 2 Division of Medical Genetics & Metabolism, University of Texas Medical Branch, Galveston, TX, United States; 3 Department of Pathology and Laboratory Medicine, University of Texas San Antonio, San Antonio, TX, United States

**Keywords:** aneuploidy, corneal haze, genetic counseling, karyotype, microcornea, prenatal testing, shared decision making, trisomy 22

## Abstract

**Introduction:**

: Trisomy 22 is identified in 2%–5% of spontaneous abortions across various populations, making it the second most common trisomy in spontaneous abortions. Liveborn patients with complete trisomy 22 are rare and present with variable clinical findings.

**Methods:**

A detailed clinical report of a term female neonate with complete trisomy 22 was compiled after obtaining informed consent from her mother.

**Results:**

The neonate was born via spontaneous vaginal delivery, and resuscitation included intubation after multiple attempts. She presented with multiple congenital anomalies, including a single umbilical artery, microcephaly, dysmorphic facial features, absent red reflex, corneal haze, irregular pupil, microcornea, imperforated anus with rectovestibular fistula, bilateral clinodactyly of the fifth digits, and bilateral distal and proximal transverse palmar creases running parallel to each other. An echocardiogram showed Tetralogy of Fallot and hypoplasia of the transverse aortic arch. Abdominal ultrasound revealed small kidneys. The electroencephalogram showed diffuse cerebral dysfunction. Head ultrasound indicated absence of the septum pellucidum. Additional complications included pneumothorax, metabolic disturbances, sepsis, and persistent thrombocytopenia. The neonate's condition deteriorated, leading to demise on the seventh day of life. Chromosome analysis confirmed 47,XX,+22.

**Discussion:**

: This case report contributes to the limited literature on complete trisomy 22, highlighting its prognosis, clinical heterogeneity, and management challenges. The constellation of anomalies observed underscores the importance of interdisciplinary collaboration and adaptability in providing optimal care. By better understanding its clinical presentation, we hope to facilitate early recognition, appropriate management, and counseling.

## 1 Introduction

The incidence of liveborn trisomy 22 is estimated to be 1 in 30,000–50,000 (1). To date, only 27 liveborn infants with complete trisomy 22 have been reported (1–4). The median survival is estimated to be 4 days (1), with the most common cause of death being respiratory insufficiency (1–3, 5, 6).

Trisomy 22 is frequently observed in spontaneous abortions and is reported as the second most common autosomal trisomy after trisomy 16 (1, 3, 7). It is found in 2%–5% of spontaneous abortions across all populations (8), most often during the first trimester (9). As with most aneuploidies, increased maternal age is a risk factor, with non-disjunction errors most likely occurring in maternal meiosis one. More female than male patients with this syndrome have been described (10–12), most likely due to the higher incidence of pregnancy loss among male fetuses, a pattern observed in other aneuploidies as well (13, 14).

Because complete trisomy 22 is a lethal condition, the available literature is limited, and the phenotypic spectrum has been largely characterized through reports of mosaic trisomy 22. Clinical features include intrauterine growth restriction, microcephaly, hydrocephalus with macrocephaly, seizures, cleft palate, webbed neck, congenital heart disease (septal defects are more commonly reported in neonates with mosaic aneuploidy, whereas anomalous great vessels are more commonly reported in neonates with complete trisomy 22), renal anomalies, anal atresia, thumb anomalies, hypotonia, and dysmorphic facies (2, 5, 6, 15–19). Dysmorphic facial features include an abnormal hairline, hypertelorism, epicanthal folds, a flat nasal bridge, low-set ears, and preauricular pits (2, 3, 16). Infants with mosaic trisomy 22 are expected to have developmental delays, and rapid deterioration with regression in developmental milestones after 4 years of age has been reported in such cases (18, 20).

Here, we report a case of complete trisomy 22 and review some of the prenatal diagnostic tools available today. Unfortunately, the results of genetic testing, and consequently the final diagnosis, became available only after the patient's death, preventing their use for clinical management. A rapid diagnosis and understanding of the prognosis are crucial for providing appropriate guidance to parents. This involves engaging in conversations with the family about expected outcomes, potential medical or surgical challenges, and management strategies.

Informed consent for publication of this case report, including clinical data, photographs, and karyotype results, was obtained from the patient's mother after the patient's death. All identifying information has been removed to protect the privacy of the patient and family.

## 2 Case presentation

A female neonate was born at 37 weeks and 1 day via spontaneous vaginal delivery to a 28-year-old mother with one prior spontaneous miscarriage, with no genetic testing performed on the products of conception. The patient was the product of a naturally conceived pregnancy. The father was 28 years old, and the parents are non-consanguineous. There were no reported teratogenic exposures.

Prenatal ultrasounds showed a large ventricular septal defect, an abnormal aorta, dilated small bowel, a pelvic right kidney, and micrognathia. A non-invasive prenatal testing (NIPT) result was inconclusive but suggested a possible sex chromosome anomaly involving the X chromosome, predicted to be of placental/fetal origin, with low risk for trisomy 13, 18, and 21; triploidy; and 22q11.2 microdeletion syndrome. Abnormalities in other chromosomes were not tested. No invasive prenatal genetic tests were obtained per the family's preference. Family history was negative for prior birth defects and neonatal deaths.

The patient was small for gestational age, being under the 10th percentile for weight (1,750 g), length (42.5 cm), and head circumference (30 cm). The Apgar scores were 2, 4, and 4 at 1, 5, and 10 min, respectively. At delivery, the infant was noted to have cyanosis, a weak cry, and was unresponsive to stimulation. Despite bagging attempts at 2 min of life, there was no improvement in heart rate or oxygen saturation. Intubation was unsuccessfully attempted five times; chest compressions were initiated, and epinephrine was given. A laryngeal mask airway was successfully placed, resulting in improved oxygen saturation. The infant was transferred from the radiant warmer to an isolette at 21 min of life. She was subsequently intubated using a 2.9 mm rigid bronchoscope with a 3.0 mm endotracheal tube. It was noted that the epiglottis and airway were extremely anterior and difficult to visualize. Rigid tracheoscopy revealed a patent trachea and mainstem bronchi without apparent tracheoesophageal fistula. She received one dose of surfactant and inhaled nitric oxide without improvement. Ventilation remained challenging despite high settings. She developed bilateral pneumothorax on day 1 of life, requiring bilateral chest tubes. She also developed severe hypoglycemia. The physical examination and further imaging revealed several congenital anomalies included in Tables 1, 2 and Figures 1, 2.

**Table 1:** Summary of clinical findings and diagnostic investigations in our patient.

System	Imaging findings
Head and craniofacial	• Micrognathia and flat midface (noted on prenatal ultrasound)
Neurological	• Cerebral ultrasound revealed the absence of the septum pellucidum and a small cystic change in the left caudothalamic groove, possibly resulting from prior hemorrhage or chord plexus cyst • A 3-h neonatal video electroencephalogram conducted on the fourth day of life showed: - Diffuse cerebral dysfunction, with no distinction between awake and sleep states - Moderate voltage bursts, primarily delta activity, alternating with briefer, desynchronized lower voltage interburst periods composed mostly of mixed theta and delta activity - No seizures
Cardiovascular	• Prenatal ultrasound done at 20 weeks and 1 day of gestation showed a large ventricular septal defect and an abnormal aorta • An echocardiogram performed within 24 h of birth revealed the following: - Tetralogy of Fallot - Large secundum atrial septal defect (ASD) - Large patent ductus arteriosum - Dilated main pulmonary artery - Thickened pulmonary, aortic, and tricuspid valves - Mild hypoplasia of the transverse aortic arch without evidence of aortic coarctation
Gastrointestinal	• Prenatal ultrasound showed dilated small bowel • abdominal ultrasound revealed moderate ascites
Genitourinary	• Prenatal ultrasound showed pelvic right kidney • Abdominal ultrasound revealed low-lying, small-for-age kidneys with normal parenchymal echotexture

**Table 2:** Summary of published cases of non-mosaic trisomy 22 fetuses identified in the literature over the past 50 years, to the best of our knowledge.

Citation	Reported phenotype	Pregnancy/neonatal outcome
Bacino et al. (16)	Describes 2 cases of confirmed non-mosaic Trisomy 22: General: fetal growth restriction (2/2) Head and craniofacial: ocular hypertelorism (2/2), epicanthal folds (1/2), midface hypoplasia (2/2), short nose with broad flat nasal bridge (2/2), microtia (1/2), small jaw (1/2), cleft palate (1/2), short broad neck with marked webbing (2/2) Chest and lungs: narrow chest, wide-spaced nipples (2/2) Cardiovascular: left superior vena cava emptying into the coronary sinus (1/2), hypoplastic aortic arch (1/2), common pulmonary vein connecting with the lower lobes of the lungs and emptying into the left atrium (1/2), endocardial cushion the defect with absent atrial septum and membranous ventricular septal defect (2/2), hypoplastic right heart (1/2) Gastrointestinal: annular pancreas Genitourinary: hypoplastic kidneys with bilateral bifid ureters (1/2), empty scrotal sac, short penis with hypospadias (1/2). Normal genitalia (1/2) Musculoskeletal: limited forearm extension (2/2), single transverse palmar crease on the right, limited knee extension, equinovalgus rocker-bottom feet, short 1st and 5th toes with a wide space between the 1st and 2nd toes (1/2)	One pregnancy was terminated at 31 weeks, and the other one resulted in a live birth that decreased at 2 months of age
Trevisan et al. (21)	General: fetal growth restriction, oligohydramnios Head and craniofacial: frontal bossing, upslanting eyelids, low-set ears, flat nose, wide mouth, microretrognathia, thickened nuchal fold Cardiovascular: double outlet right ventricle, single umbilical artery Chest: hypoplastic thymus Musculoskeletal: talipes, fixed limbs and hands flexed	Miscarriage at 19 weeks
Ma et al. (22)	General: fetal growth restriction Head and craniofacial: flat facial profile Cardiovascular: ventricular septal defect Neurological: long-sectional (long-segment) spina bifida occulta Gastrointestinal: imperforated anus Genitourinary: abnormal bilateral kidney structures Musculoskeletal: rocker-bottom right foot	Terminated at 24 weeks (2nd trimester)
Minella et al. (23)	Describes four cases of confirmed non-mosaic Trisomy 22: General: fetal growth restriction (1/4) Head and craniofacial: persistent nuchal thickening (1/3) Cardiovascular: Tetralogy of Fallot (1/4) and atrioventricular septal defect (2/4), pulmonary artery stenosis (1/4), single umbilical artery (1/4) Gastrointestinal: hepato-splenomegaly (1/4), intestinal hyperechogenicity (1/4)	Medical termination of pregnancy in 1st and 2nd trimester
Sepulveda et al. (24)	Describes three cases of confirmed non-mosaic Trisomy 22: General: fetal growth restriction (1/3), subcutaneous edema (2/3) Head and craniofacial: microcephaly, abnormal head shape, hypertelorism, downward-slanting palpebral fissure, low-set ears, flat midface, down-turned mouth, cleft palate (2/3), short neck (2/3) Chest and lungs: bilateral pleural effusions (2/3), broad chest (1/3), agenesis of the diaphragm (1/3) Cardiovascular: atrioventricular septal defect (1/3), interventricular septal defect (1/3), single umbilical artery (1/3) Genitourinary: blind-ended vaginal canal (1/3), rudimentary unicornuate uterus (1/3), ambiguous genitalia (1/3), left renal agenesis (2/3), multicyclic kidney (1/3) Musculoskeletal: fixed flexion of the left elbow (1/3), left talipes (1/3), bilateral short fifth finger (1/3), hypoplastic limbs (1/3)	One pregnancy was terminated at 19 weeks, and two ended in fetal demise at 23 and 26 weeks
Mokate et al. (25)	Describes 3 cases of confirmed non-mosaic Trisomy 22: General: fetal growth restriction (1/2), oligohydramnios (1/2) Head and craniofacial: dolichocephaly (1/2), brachycephaly (1/2), hypertelorism (1/2), low-set ears with preauricular dimples and tags (1/2), broad flat nasal bridge (1/2), flat facial profile (1/2), cleft lip and palate (1/2), long philtrum (1/2), micrognathia (1/2) Cardiovascular: possible ventricular septal defect (1/2) Genitourinary: normal external genitalia (1/2) Musculoskeletal: talipes (2/2), camptodactyly with flexion deformities at 3rd, 4th, and 5th proximal interphalangeal joints, two creases on the left hand, and one on the right hand (1/2), skin syndactyly bilaterally between fingers 2 to 5 (1/2)	One stillborn delivered at 40 weeks and the other pregnancy was terminated at 20 weeks (2nd trimester)
Morrison et al. (26)	Oligohydramnios was present. Detailed postmortem examination of the fetus did not reveal any structural abnormalities.	Stillborn delivered at 39 weeks
Harding et al. (27)	General: fetal growth restriction Head and craniofacial: bilateral cleft lip Genitourinary: left kidney agenesis Musculoskeletal: reduced femur-to-foot length ratio	Pregnancy was terminated (unknown trimester)
Our patient	General: Small for gestational age Head and craniofacial: square facies with tall and broad forehead, hypertelorism, downward slanting palpebral fissures, microphthalmia, absent red reflex, corneal haze on the right along with an irregular pupil, microcornea on the left, wide nasal bridge, micrognathia, deep nasojugal creases, bilateral microtia with periauricular tags (Figures 2, 3), neck webbing with redundant nuchal skin and edema Neurological: microcephaly, wide anterior fontanelle, absence of the septum pellucidum Cardiovascular: Tetralogy of Fallot, large secundum ASD, large PDA, dilated main pulmonary artery, thickened pulmonary, aortic, and tricuspid valves, single umbilical artery Thorax: widely spaced nipples Gastrointestinal: imperforated anus Genitourinary: low-lying and small-for-age kidneys, typical female external genitalia Musculoskeletal: pedal edema, bilateral clinodactyly of the fifth digits, bilateral distal and proximal transverse palmar creases running in parallel across the full width of the palm Skin: hyperpigmentation in thoracic and lumbar back	Deceased at 7 days of life

Publications describing non-mosaic trisomy 22 in association with other genetic abnormalities were excluded.

**Figure 1:**
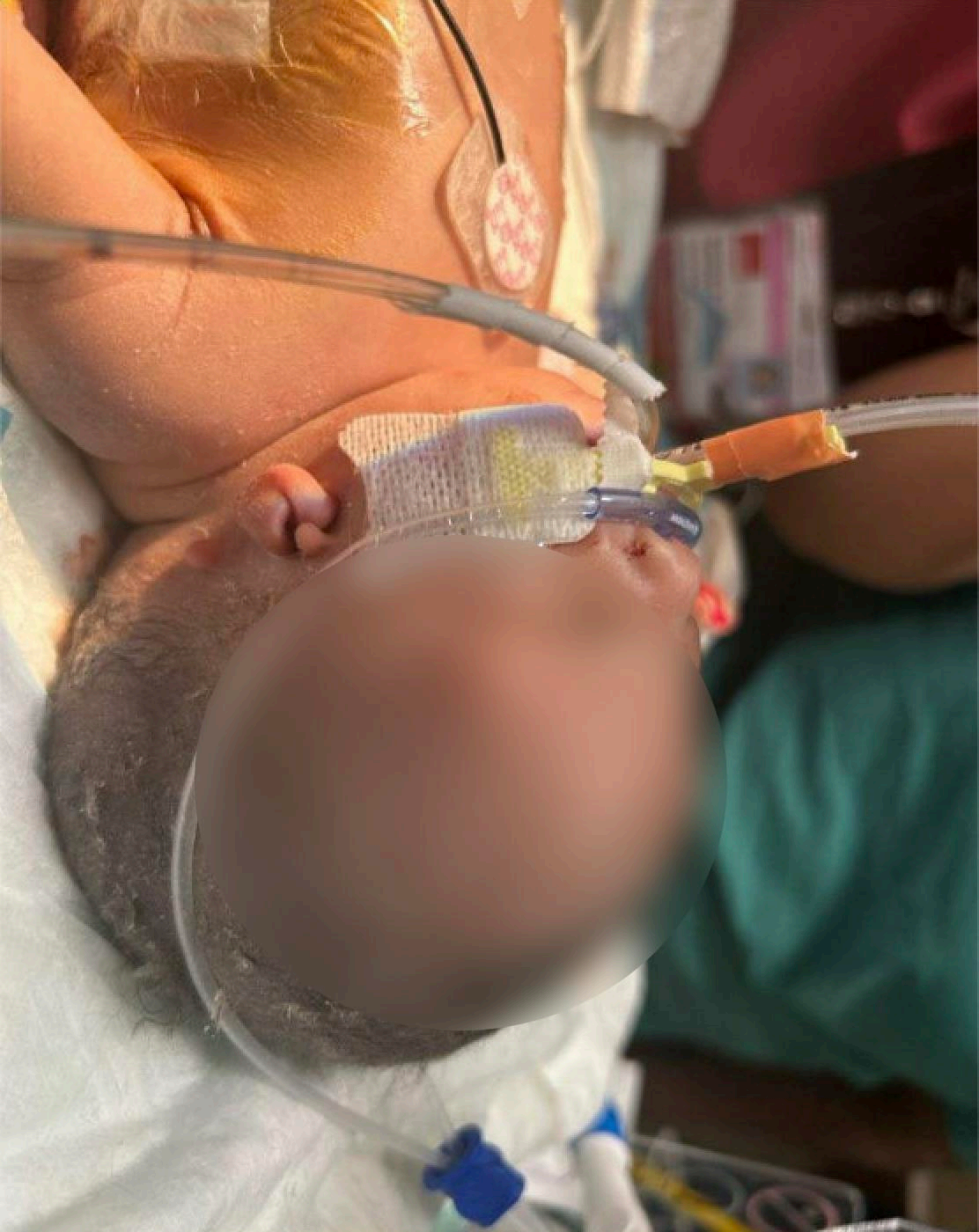
A lateral view of a neonate's face demonstrating a flat midface, a wide nasal bridge, and left ear abnormalities, including microtia and an ear tag. The infant is intubated and receiving supportive care, with visible medical adhesive patches and monitoring devices attached.

**Figure 2:**
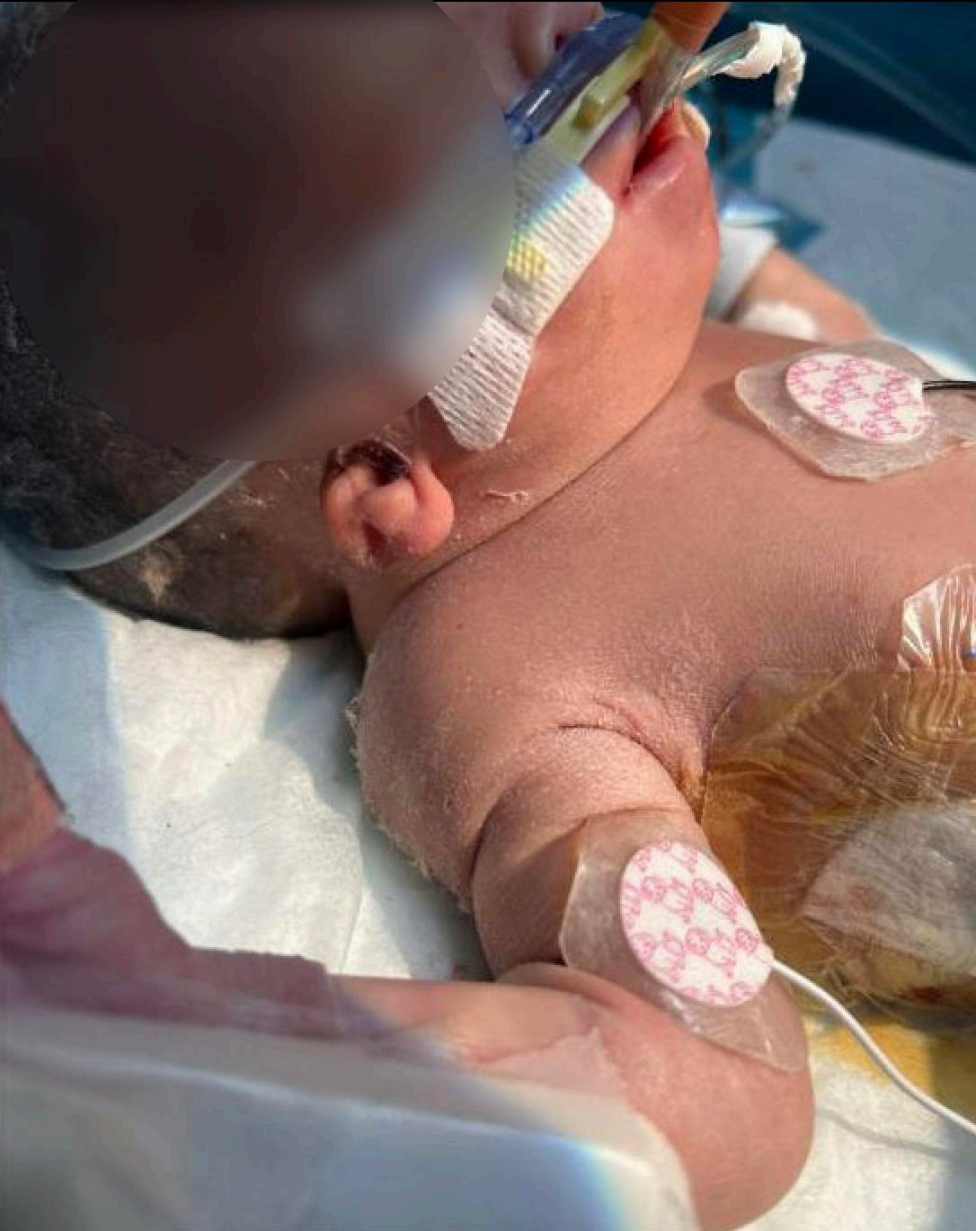
Close-up view of the patient's right ear.

From an infectious disease standpoint, the patient received a 5-day course of ampicillin and ceftazidime due to maternal chorioamnionitis. On the fourth day of life, the patient was diagnosed with coagulase-negative *Staphylococcus sepsis*, and vancomycin was started. Our patient experienced persistent thrombocytopenia requiring multiple platelet transfusions and impaired thermoregulation necessitating warmer therapy.

By the fourth day of life, evidence of pulmonary hypertension emerged, with predominantly right-to-left shunting across the patent ductus arteriosum. The patient required daily hydrocortisone for hypotension. On the sixth day of life, the patient's hypotension worsened, requiring epinephrine and calcium chloride. On the seventh day of life, the decision was made to withdraw life-sustaining therapies, leading to the discontinuation of vasopressors and neuromuscular blockade. The endotracheal tube was removed, and the patient passed away approximately 1.5 h later. A mitogen-stimulated peripheral blood chromosome study showed trisomy 22 in all 50 cells analyzed, with the karyotype 47,XX,+22 (Figure 3). Autopsy was not performed.

**Figure 3:**
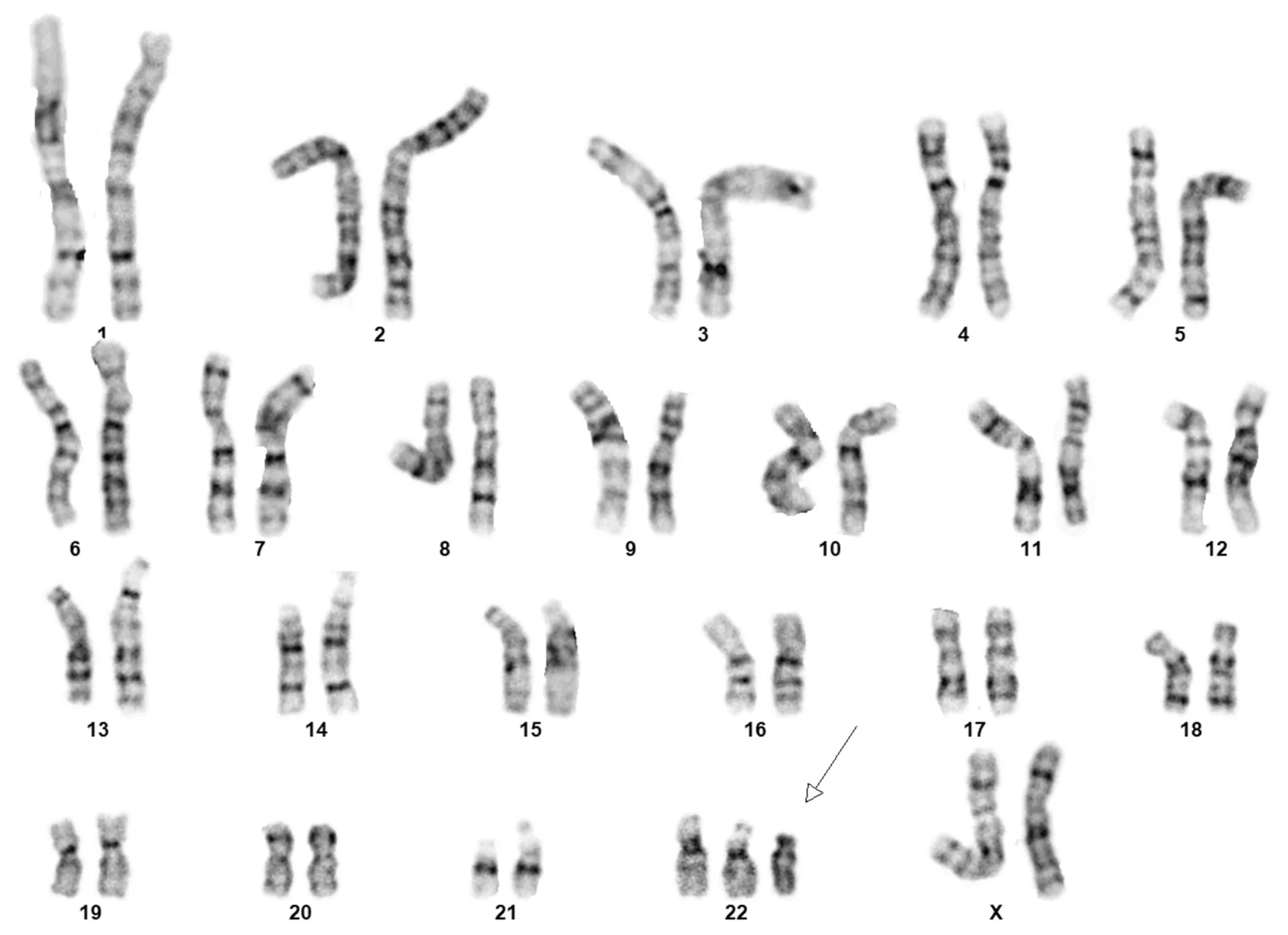
Karyotype 47,XX,+22 obtained from a mitogen-stimulated peripheral blood chromosome study. The extra chromosome 22, indicative of trisomy 22, is marked with an arrow.

## 3 Discussion

Trisomy 22 is the second most common trisomy in spontaneous abortions, with trisomy 16 occurring more frequently (1, 3, 7). Hall et al. described trisomy 22 as the most commonly occurring trisomy due to maternal non-disjunction during meiosis one (28). While many cases of mosaic trisomy 22 have been reported (as noted in Table 3), complete trisomy 22 live births are rare due to an adverse prognosis, with a median survival of 4 days (1).

**Table 3:** Summary of published cases of mosaic trisomy 22 identified in the literature over the past 50 years, to the best of our knowledge.

Publication	Tissue and karyotype	Clinical phenotype	Developmental and cognitive outcome	Survival status
Chen et al. (10)	Cord/Placenta: 47,XY,+22/46,XY (9–37.5% mosaicism); blood/amniocytes: 46,XY	General: small for gestational age Head and craniofacial: cleft palate Gastrointestinal: imperforated anus	Not assessed	Liveborn delivered at 36 weeks
Abdelgadir et al. (15)	Cord: 47,XX,þ22 in 50% of cells. Cardiomyocytes from the right atrial appendage obtained during her cardiac surgery: 47,XX,þ22 (5)/46,XX(25). Fibroblast: 47,XX,þ22 (6)/46,XX44	General: small for gestational age Head and craniofacial: low anterior hairline, epicanthal folds, right periauricular pit, downturned mouth Cardiovascular: patent ductus arteriosus and tetralogy of Fallot Gastrointestinal: anteriorly displaced anus Musculoskeletal: bilateral 5th finger clinodactyly, broad thumbs, leg length discrepancy	Mild gross motor delay	Alive at 7 years old
Trevisan et al. (21)	Placenta: [47,XY + 22 (95)/46, XY5] Cord: 46,XY Skin biopsy: 3% mT22 mosaicism Second skin biopsy: 13% mT22 mosaicism	General: small for gestational age and severely growth delay during childhood Head and craniofacial: high forehead, protruding ears with simplified helix, right eye ptosis, epicanthal folds, sparse eyebrows on the lateral third, long beaked nose, midface hypoplasia, high arched palate with dental crowding, webbed neck. Normal audiological screening and fundoscopic exam Neurological: normal brain MRI Cardiovascular: atrial septal defect and persistence of superior vena cava in the coronary sinus Gastrointestinal: anteriorized imperforated anal orifice Genitourinary: cryptorchidism, fan-shaped foreskin Musculoskeletal: hypotonia, severe kyphosis, postural bilateral talipes, bilateral clinodactyly	Mildly delayed neurodevelopment, oppositional behavior, and difficulties in social interactions	Alive at 17 years old
Lewis et al. (38) and Crowe et al. (45)	Fibroblasts: 47,XY,+22[28]/46,XY[2]; Lymphocytes: 47,XY,+22[4]/46,XY[96]	General: small for gestational age and severely growth delay during childhood Head and craniofacial: dolichocephaly, bilateral preauricular pits, severe hearing loss, epicanthal folds, midface hypoplasia, notched upper central incisors Neurological: intractable seizures in neonatal period with normal electroencephalogram, hypotonia in childhood Cardiovascular: patent ductus arteriosus Genitourinary: undescended testicles Musculoskeletal: hemiatrophy of right arm	Moderate intellectual disability (IQ≤55) Processing speed strengths, math/motor delays	Alive at 14 years old
Leclercq et al. (39)	Case 1: Fibroblast: 47,XX,+22 (6)/46,XX(94) Lymphocytes: 46,XX(100) Case 5: Fibroblast: 47,XX,+22 (33)/46,XX(67) Lymphocytes: 46, XX	Case 1: normal exam Case 5: General: small for gestational age Head and craniofacial: high frontal hairline, prominent forehead, hypertelorism, down-slanted palpebral fissures, low-set large ears, anteverted nares, long philtrum Neurological: cortical atrophy Cardiovascular: ventricular septal defect Musculoskeletal: facial and body asymmetry, left coxa valga, distal phalange agenesis of the left hand and foot	Case 1: normal cognitive, behavioral, and physical development Case 5: not assessed	Case 1: alive at 4 years old Case 5: alive at 9 months
Kalayinia et al. (40)	Lymphocytes: 47,XY,+22[6]/46,XY[44] (12% mosaicism)	General: small for gestational age and severe growth delay during childhood Head and craniofacial: facial asymmetry, small and rotated right ear, bilateral hearing loss, flattened nasal bridge, cleft palate, dental anomalies Neurological: normal brain MRI Cardiovascular: atrial septal defect Genitourinary: hydronephrosis, hypospadias, unilateral cryptorchidism, ambiguous genitalia Musculoskeletal: general hypotrophy	Normal intelligence; delayed motor milestones and delayed speech onset	Alive at 4 years old
He et al. (41)	Lymphocytes: 47,XY,+22	General: small for gestational age Head and craniofacial: two preauricular fistulas, flat nasal bridge, micrognathia Cardiovascular: tetralogy of Fallot Gastrointestinal: micropenis Genitourinary: micropenis	Not assessed	Family requested discharge against medical advice on day of life 5, and the patient died 1 month after
Wang et al. (42)	Amniocytes: 47,XX,+22[6]/46,XX[11]; Fibroblasts: 47,XX,+22[16]/46,XX[5]; Blood: 46,XX	General: small for gestational age Head and craniofacial: hypertelorism, bilateral infraorbital creases, right preauricular pit, crumpled left ear helix, flat nasal bridge, thin upper and lower lip, narrow philtrum, high-arched palate, thick nuchal fold Cardiovascular: cardiomyopathy Genitourinary: hypoplastic labia majora with hemiatrophy toward the right side, enlarged clitoris Skin: hemangiomas on eyelids, nose, and forehead Musculoskeletal: tapered fingers, hypoplastic finger and toenails, bilateral clinodactyly of the fifth finger, a crease extending laterally from the second webspace of the toe	Not assessed	Liveborn delivered at 35 weeks
De Pater et al. (43)	Fibroblasts: 47,XX, +22 (7)/46,XX(25) karyotype	General: small for gestational age and severe growth delay during childhood Head and craniofacial: epicanthal folds, upslanted palpebral fissures, proptosis, broad nasal bridge, short nose, long and smooth philtrum, low-set ears with preauricular pit bilaterally, micrognathia Cardiovascular: ventricular septal defect Back: sacral dimple Musculoskeletal: hypotonia, clinodactyly	Delayed motor development	Alive at 19 months old
Mazza et al. (44)	Amniocytes: 47, XY, +22 (3)/46,XY6); Placenta: 47, XY, +22 (95%−100%); Fibroblasts: 47, XY, +22 (3%–4%); Lymphocytes: 46, XY	General: small for gestational age and severe growth delay during childhood Head and craniofacial: wide and frontally bossed forehead, ptosis of the right eye, low-set ears with long lobes, large and flat nasal root, anteverted nostrils. Normal fundal examination and auditory evoked potentials Neurological: normal brain MRI Cardiovascular: ostium secundum and persistence of the superior vena cava in the coronary sinus Gastrointestinal: anterior anus Genitourinary: cryptorchidism Musculoskeletal: hypotonia	Delayed motor and speech development	Alive at 31 months old

Publications describing mosaic trisomy 22 in association with other genetic abnormalities were excluded.

Prenatal ultrasonography plays a fundamental role in the detection of trisomy 22. Findings are variable but, in most cases, include a single umbilical artery, microcephaly, cardiac defects, and renal abnormalities. However, these findings are non-specific, and a definitive diagnosis currently requires invasive genetic testing methods such as chorionic villus sampling or amniocentesis (21). In our patient, prenatal ultrasonography demonstrated a ventricular septal defect, one of the most frequently reported cardiac anomalies in trisomy 22, along with an aortic abnormality, which has been described in a single prior report (16). The micrognathia, flat midface, and renal abnormalities identified prenatally are also consistent with previously described features of trisomy 22. Notably, the dilated small bowel observed on prenatal ultrasonography has not been previously reported in association with trisomy 22.

The prenatal diagnostic landscape has evolved substantially in recent years and now includes studies like NIPT, chromosomal microarray analysis (CMA), and next-generation sequencing technologies. Both the American College of Medical Genetics and Genomics (ACMG) and the American College of Obstetricians and Gynecologists (ACOG) recommend that NIPT be made available to all pregnant patients, regardless of maternal age or risk. While NIPT offers a promising screening tool, most laboratories, including the one that was used for this patient's NIPT, only report abnormalities in chromosomes 13, 18, 21, and the sex chromosomes and do not screen for rarer aneuploidies like trisomy 22. This testing relies on placental DNA rather than fetal DNA, which can limit its ability to fully detect fetal genetic conditions, necessitating confirmatory invasive tests (29–31). In our patient, the NIPT showed an increased risk for sex chromosome aneuploidy. The prenatal testing laboratory was unable to provide any further clarification on the possible sex chromosome aneuploidy that the screen was suspicious for or any additional information about the screening result when provided with the patient's chromosome analysis results. Thus, we suspect this was a false-positive screening result unrelated to the confirmed diagnosis of trisomy 22. However, our patient did not have a CMA completed, so we did not rule out a copy number variant on her sex chromosomes that was too small to be detected by chromosome analysis and may have led to the positive NIPT.

CMA is recommended by the ACOG and ACMG as a first-tier diagnostic test for fetuses with structural anomalies, as it identifies all aneuploidies detected by karyotype while additionally detecting submicroscopic copy number variants in approximately 6% of cases with a normal karyotype (32, 33). In addition, whole exome sequencing (WES) and whole-genome sequencing (WGS) can provide superior diagnostic yields compared to CMA alone (34). While ACOG and ACMG currently recommend WES or WGS only when other tests have failed to arrive at a diagnosis in a fetus with multiple congenital anomalies, these technologies can provide earlier and more precise characterization of complex fetal phenotypes.

At birth, our patient exhibited a constellation of clinical features consistent with previously reported cases of trisomy 22 (35). Notably, our case highlighted additional findings, including corneal haze, microcornea, and absence of the septum pellucidum, which have not been previously reported to our knowledge. The most common eye abnormality reported in mosaic and complete trisomy 22 is coloboma (36, 37). Table 2 shows additional details and a comparison of our patient to other previously reported cases of trisomy 22.

The diagnosis was established by conventional chromosome analysis after the patient had passed, causing significant uncertainty for both the multidisciplinary care team and the family, who were required to make complex medical decisions without adequate time to fully understand or process the underlying condition and its prognosis. Without a clear understanding of the underlying condition and its prognosis, the family had to make complex medical decisions within a limited timeframe and in the context of rapidly evolving clinical deterioration. During the patient's course, multiple invasive interventions were required, including intubation after several attempts, high levels of ventilatory support, chest tube placement, and platelet transfusions. Ultimately, the family faced the difficult decision to withdraw life-sustaining therapies for their child. The necessity of prenatal diagnosis is critical in cases of trisomy 22. Given the high likelihood of fetal demise or limited life expectancy, preparing the family for potential outcomes and collaboratively planning interventions that align with the family's values and goals becomes essential from the point of initial diagnosis.

## 4 Conclusion

Our case report contributes to the limited body of literature on complete trisomy 22, highlighting the clinical heterogeneity and challenges associated with this rare genetic disorder. As access to prenatal genomic technologies continues to improve, earlier and more accurate diagnosis is essential to facilitate informed parental decision-making, optimize multidisciplinary perinatal planning, avoid unnecessary diagnostic studies and interventions, and provide timely genetic counseling.

## Data Availability

The datasets presented in this article are not readily available because of ethical and privacy restrictions. Requests to access the datasets should be directed to the corresponding author.
