# Supplementary material for: Liveborn trisomy 22: a case report
Source: Front Pediatr. 2026 Oct 14;14:1920011. doi: 10.3389/fped.2026.1920011 (PMC13713506; doi:10.3389/fped.2026.1920011)
Supplement: Supplementary file 1 [file Datasheet1.pdf]

PUBLICATION	TISSUE & KARYOTYPE	CLINICAL PHENOTYPE	DEVELOPMENTAL & COGNITIVE	SURVIVAL STATUS
**Chen et al. (2017)**<br>[10]	**Cord/Placenta:** 47,XY,+22/46,XY (9–37.5% mosaicism)<br>**Amniocytes:** 46,XY	**General:** Small for gestational age (SGA)<br>**Head & Craniofacial:** Cleft palate<br>**Gastrointestinal:** Imperforate anus	*Not assessed*	**Liveborn**<br>Delivered at 36 weeks
**Abdelgadir et al. (2013)**<br>[15]	**Cord:** 47,XX,+22 (50%)<br>**Cardiomyocytes:** 47,XX,+22[5]/46,XX[25]<br>**Fibroblasts:** 47,XX,+22[6]/46,XX[44]	**General:** Small for gestational age<br>**Head & Craniofacial:** Low anterior hairline, epicanthal folds, right periauricular pit, downturned mouth<br>**Cardiovascular:** Patent ductus arteriosus (PDA), Tetralogy of Fallot<br>**Gastrointestinal:** Anteriorly displaced anus<br>**Musculoskeletal:** Bilateral 5th finger clinodactyly, broad thumbs, leg length discrepancy	Mild gross motor delay	**Alive**<br>7 years old
**Trevisan et al. (2024)**<br>[22]	**Placenta:** 47,XY,+22[95]/46,XY[5]<br>**Cord:** 46,XY<br>**Skin Biopsy 1:** 3% mT22<br>**Skin Biopsy 2:** 13% mT22	**General:** Small for gestational age, severe postnatal growth delay<br>**Head & Craniofacial:** High forehead, protruding ears, right eye ptosis, epicanthal folds, sparse eyebrows, long beaked nose, midface hypoplasia, high arched palate, webbed neck<br>**Neurological:** Normal brain MRI<br>**Cardiovascular:** Atrial septal defect (ASD), persistent SVC in coronary sinus<br>**Gastrointestinal:** Anteriorized imperforated anal orifice<br>**Genitourinary:** Cryptorchidism, fan-shaped foreskin<br>**Musculoskeletal:** Hypotonia, severe kyphosis, postural bilateral talipes, clinodactyly	Mildly delayed neurodevelopment, oppositional behavior, social interaction difficulties	**Alive**<br>17 years old
**Lewis et al. (2007)**<br>**Crowe et al. (1997)**<br>[32]	**Fibroblasts:** 47,XY,+22[28]/46,XY[2]<br>**Lymphocytes:** 47,XY,+22[4]/46,XY[96]	**General:** Small for gestational age, severe postnatal growth delay<br>**Head & Craniofacial:** Dolichocephaly, preauricular pits, severe hearing loss, epicanthal folds, midface hypoplasia, notched upper central incisors<br>**Neurological:** Intractable neonatal seizures (normal EEG), childhood hypotonia<br>**Cardiovascular:** Patent ductus arteriosus (PDA)<br>**Genitourinary:** Undescended testicles<br>**Musculoskeletal:** Hemiatrophy of right arm	Moderate intellectual disability (IQ ≤ 55), processing speed strengths, math & motor delays	**Alive**<br>14 years old
**Leclercq et al. (2010)**<br>[33]	<u>**Case 1**</u><br>**Fibro:** 47,XX,+22[6]/46,XX[94]<br>**Lymph:** 46,XX[100]<br><u>**Case 5**</u><br>**Fibro:** 47,XX,+22[33]/46,XX[67]<br>**Lymph:** 46,XX	<u>**Case 1**</u><br>Normal physical exam<br><u>**Case 5**</u><br>**General:** Small for gestational age<br>**Head & Craniofacial:** High frontal hairline, prominent forehead, hypertelorism, down-slanted palpebral fissures, low-set large ears, anteverted nares, long philtrum<br>**Neurological:** Cortical atrophy<br>**Cardiovascular:** Ventricular septal defect (VSD)<br>**Musculoskeletal:** Facial/body asymmetry, left coxa valga, distal phalange agenesis of left hand & foot	<u>**Case 1**</u><br>Normal cognitive & physical dev<br><u>**Case 5**</u><br>*Not assessed*	<u>**Case 1**</u><br>**Alive** (4 yrs)<br><u>**Case 5**</u><br>**Alive** (9 mos)
**Kalayinia et al. (2019)**<br>[34]	**Lymphocytes:** 47,XY,+22[6]/46,XY[44] (12% mosaicism)	**General:** Small for gestational age, severe postnatal growth delay<br>**Head & Craniofacial:** Facial asymmetry, small/rotated right ear, hearing loss, flat nasal bridge, cleft palate, dental anomalies<br>**Neurological:** Normal brain MRI<br>**Cardiovascular:** Atrial septal defect (ASD)<br>**Genitourinary:** Hydronephrosis, hypospadias, unilateral cryptorchidism, ambiguous genitalia<br>**Musculoskeletal:** General hypotrophy	Normal intelligence; delayed motor milestones and speech onset	**Alive**<br>4 years old
**He et al. (2015)**<br>[35]	**Lymphocytes:** 47,XY,+22	**General:** Small for gestational age<br>**Head & Craniofacial:** Two preauricular fistulas, flat nasal bridge, micrognathia<br>**Cardiovascular:** Tetralogy of Fallot<br>**Genitourinary:** Micropenis	*Not assessed*	**Deceased**<br>DAMA day 5; died 1 mo

PUBLICATION	TISSUE & KARYOTYPE	CLINICAL PHENOTYPE	DEVELOPMENTAL & COGNITIVE	SURVIVAL STATUS
**Wang et al. (2007)**<br>[36]	**Amniocytes:** 47,XX,+22[6]/46,XX[11]<br>**Fibroblasts:** 47,XX,+22[16]/46,XX[5]<br>**Lymphocytes:** 46,XX	**General:** Small for gestational age<br>**Head & Craniofacial:** Hypertelorism, infraorbital creases, preauricular pit, crumpled ear helix, flat nasal bridge, thin lips, narrow philtrum, high-arched palate, thick nuchal fold<br>**Cardiovascular:** Cardiomyopathy<br>**Genitourinary:** Hypoplastic labia majora with right hemiatrophy, enlarged clitoris<br>**Skin:** Hemangiomas on eyelids, nose, and forehead<br>**Musculoskeletal:** Tapered fingers, hypoplastic nails, clinodactyly, lateral toe crease	*Not assessed*	**Liveborn**<br>Delivered at 35 weeks
**De Pater et al. (1997)**<br>[37]	**Fibroblasts:** 47,XX,+22[7]/46,XX[25]	**General:** Small for gestational age, severe postnatal growth delay<br>**Head & Craniofacial:** Epicanthal folds, upslanted palpebral fissures, proptosis, broad nasal bridge, short nose, long/smooth philtrum, low-set ears, preauricular pits, micrognathia<br>**Cardiovascular:** Ventricular septal defect (VSD)<br>**Back:** Sacral dimple<br>**Musculoskeletal:** Hypotonia, clinodactyly	Delayed motor development	**Alive**<br>19 months old
**Mazza et al. (2010)**<br>[38]	**Amniocytes:** 47,XY,+22[3]/46,XY[6]<br>**Placenta:** 47,XY,+22 (95–100%)<br>**Fibroblasts:** 47,XY,+22 (3–4%)<br>**Lymphocytes:** 46,XY	**General:** Small for gestational age, severe postnatal growth delay<br>**Head & Craniofacial:** Wide/frontally bossed forehead, right eye ptosis, low-set ears with long lobes, large flat nasal root, anteverted nostrils<br>**Neurological:** Normal brain MRI<br>**Cardiovascular:** Ostium secundum, persistent SVC in coronary sinus<br>**Gastrointestinal:** Anterior anus<br>**Genitourinary:** Cryptorchidism<br>**Musculoskeletal:** Hypotonia	Delayed motor and speech development	**Alive**<br>31 months old
