# Supplementary material for: Liveborn trisomy 22: a case report
Source: Front Pediatr. 2026 Oct 14;14:1920011. doi: 10.3389/fped.2026.1920011 (PMC13713506; doi:10.3389/fped.2026.1920011)
Supplement: Supplementary file 2 [file Datasheet2.pdf]

| System | Imaging findings |
|---|---|
| **Head and craniofacial** | • micrognathia and flat midface (noted on prenatal ultrasound) |
| **Neurological** | • cerebral ultrasound revealed the absence of the septum pellucidum and a small cystic change in the left caudothalamic groove, possibly resulting from prior hemorrhage or cord plexus cyst<br>• a 3-hour neonatal video electroencephalogram conducted on the fourth day of life showed:<br>– diffuse cerebral dysfunction, with no distinction between awake and sleep states<br>– moderate voltage bursts, primarily delta activity, alternating with briefer, desynchronized lower voltage interburst periods composed mostly of mixed theta and delta activity<br>– no seizures |
| **Cardiovascular** | • prenatal ultrasound done at 20 weeks and 1 day of gestation showed a large ventricular septal defect and an abnormal aorta<br>• An echocardiogram performed within 24 hours of birth revealed the following:<br>– Tetralogy of Fallot<br>– large secundum atrial septal defect (ASD)<br>– large patent ductus arteriosus<br>– dilated main pulmonary artery<br>– thickened pulmonary, aortic, and tricuspid valves<br>– mild hypoplasia of the transverse aortic arch without evidence of aortic coarctation |
| **Gastrointestinal** | • prenatal ultrasound showed dilated small bowel<br>• abdominal ultrasound revealed moderate ascites |
| **Genitourinary** | • prenatal ultrasound showed pelvic right kidney<br>• abdominal ultrasound revealed low-lying, small-for-age kidneys with normal parenchymal echotexture |
